# Significance of serum-soluble CD95 (Fas/APO-1) on prognosis in renal cell cancer patients

**DOI:** 10.1038/sj.bjc.6690576

**Published:** 1999-07

**Authors:** M Kimura, Y Tomita, T Imai, T Saito, A Katagiri, T Tanikawa, M Takeda, K Takahashi

**Affiliations:** Department of Urology, Niigata University School of Medicine, 1-757 Asahimachi, Niigata 951-8510, Japan

**Keywords:** soluble CD95 (Fas/APO-1), renal cell cancer, prognosis

## Abstract

Serum-soluble CD95 (sCD95) levels for 72 renal cell cancer patients were significantly higher than those of 17 healthy donors. Twenty-one of 72 patients had elevated (defined as more than mean of healthy donors + 2 s.d.) sCD95. The disease-specific survival rate was significantly lower in the elevated sCD95 group. Serum sCD95 level was shown to be an independent prognostic factor by univariate and multivariate analysis, indicating a possible significant role in determining treatment strategies. © 1999 Cancer Research Campaign


*British Journal of Cancer* (1999) **80**(10), 1648–1651
© 1999 Cancer Research Campaign
Article no. bjoc.1999.0576

### Significance of serum-soluble CD95 (Fas/APO-1) on prognosis in renal cell cancer patients

**M Kimura, Y Tomita, T Imai, T Saito, A Katagiri, T Tanikawa, M Takeda and K Takahashi**

Department of Urology, Niigata University School of Medicine, 1-757 Asahimachi, Niigata 951-8510, Japan

**Summary** Serum-soluble CD95 (sCD95) levels for 72 renal cell cancer patients were significantly higher than those of 17 healthy donors. Twenty-one of 72 patients had elevated (defined as more than mean of healthy donors + 2 s.d.) sCD95. The disease-specific survival rate was significantly lower in the elevated sCD95 group. Serum sCD95 level was shown to be an independent prognostic factor by univariate and multivariate analysis, indicating a possible significant role in determining treatment strategies.

**Keywords**: soluble CD95 (Fas/APO-1); renal cell cancer; prognosis

CD95 (Fas/Apo-1) is a 48-kDa Type I membrane protein and a prototypic member of the nerve growth factor/tumour necrosis factor receptor superfamily (Nagata, 1994). CD95, which is widely expressed in normal and malignant cells, and CD95 ligand are a set of regulatory components in the immune system. Activation of CD95 by CD95 ligand results in apoptosis of many cell types, and this process has been shown to play a critical role in the maintenance of immunological homeostasis and peripheral tolerance (Nagata, 1994).

In addition to membrane-anchored CD95, soluble CD95 (sCD95), generated by alternative mRNA splicing, can antagonize cell-surface CD95 function (Nagata, 1994). That is, serum sCD95 may suppress apoptosis of cancer cells by blocking CD95 ligand on lymphocytes and affect tumour progression. The biological activity of sCD95 secreted by osteosarcoma cells was demonstrated by the ability of osteosarcoma supernatants to protect the sensitive T-cell line Jurkat from anti-CD95-mediated apoptosis (Fellenberg et al, 1997). Elevated sCD95 has been reported in haematopoietic and non-haematopoietic tumour patients, and patients with metastasis indicated higher sCD95 levels than those with primary disease (Midis et al, 1996). However, sCD95 has not been examined in renal cell cancer (RCC) patients. Previously, we reported that functional CD95 was expressed in RCC (Tomita et al, 1996*a*). Here, we investigated serum sCD95 in RCC patients and showed a correlation between higher sCD95 value and poorer prognosis in these patients.

### MATERIALS AND METHODS

### Patients

Seventy-two patients with RCC were examined. Their mean age was 60.4 years, ranging from 34 to 81 years. All patients had undergone nephrectomy between October 1992 and March 1994. These patients had not received chemotherapeutic or immunomodulatory agents before the collection of samples. Cancer tissues were histologically graded according to the General Rule for Clinical and Pathological Studies on Renal Cell Carcinoma (Japanese Urological Association, Japanese Pathological Society and Japanese Radiological Society, 1992). The grading system includes low-grade (G1), moderate-grade (G2) and high-grade (G3) categories according to the degree of atypia of the tumour cells (Union Internationale Contre le Cancers (UICC), 1987). Histological stage was determined according to the TNM classification of malignant tumours. Survival was investigated in December 1997, and the mean observation period was 40.6 months.

*Received 29 September 1998*
*Revised 27 January 1999*
*Accepted 4 February 1999*

*Correspondence to:* M Kimura

### Examination of serum sCD95 and survival analysis

Serum samples were obtained from RCC patients before surgery. Serum sCD95 level were examined using a CD95-specific double sandwich enzyme-linked immunosorbent assay (ELISA) (soluble Fas ELISA kit; Medical & Biological Laboratories Co. Ltd., Nagoya, Japan). This assay uses CD95 antibodies against two different epitopes, one of the antibodies was polyclonal (No. 305–319 a.a) and the other monoclonal (No. 110–120 a.a) antibody recognized the extracellular domain. For comparison, the serum of 17 healthy donors was also evaluated. All serum samples were obtained with the consent of the patients and donors and stored at –80°C until analysis.

### Statistical analysis

The Mann–Whitney *U*-test was used to compare sCD95 levels with the healthy donors and the RCC patients. Chi-square tests were used to compare the sCD95 group (normal or elevated) with T stage, M grade and histological cell type. The Cox proportional hazards model was used in both univariate and the multivariate analysis. Survival curves were constructed using the Kaplan–Meier method, and differences between curves were tested using the log-rank test.

**Table 1** Serum sCD95 levels in relation to pathological factors

| | Normal sCD95 group | Elevated sCD95 group |
|---|---|---|
| ≤T2 | 35 | 13 |
| T3≤ | 13 | 7 |
| M0 | 42 | 15 |
| M1≤ | 7 | 5 |
| G1 | 20 | 7 |
| G2≤ | 26 | 13 |
| Clear cell subtype | 32 | 15 |
| Other subtypes | 12 | 5 |

Figures are for cases with RCC.

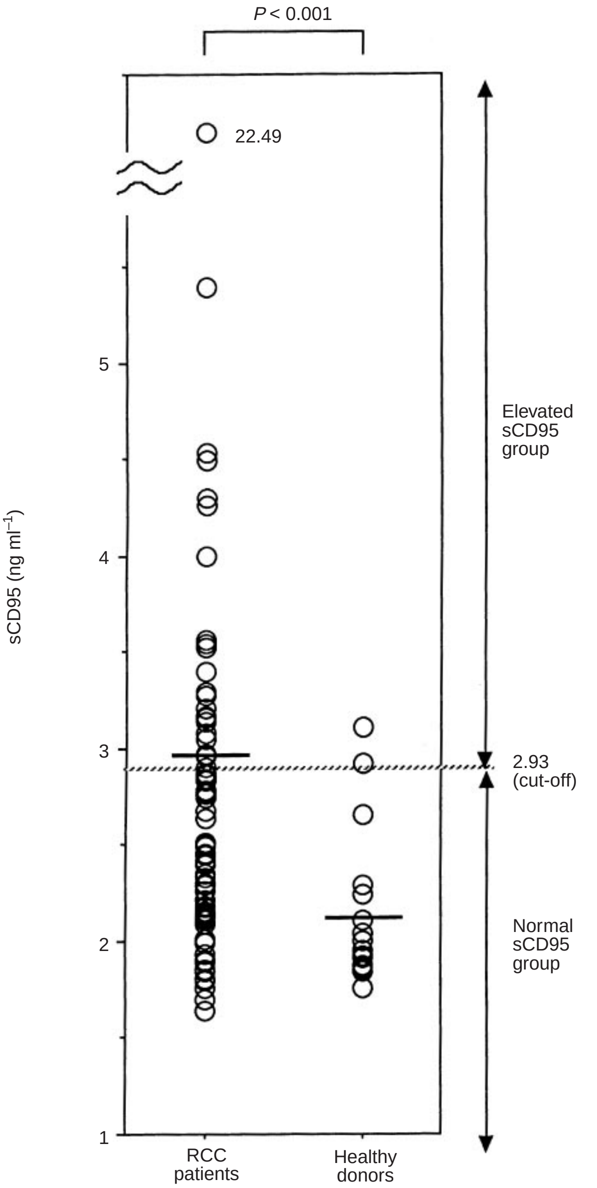

**Figure 1** sCD95 levels in serum from RCC (renal cell carcinoma) patients and healthy donors serum were examined by ELISA as described in Materials and Methods. Each ○ indicates one person. Median sCD95 levels are represented by a horizontal line across each ○ line. The dotted line indicates the cut off level defined by the mean of healthy donors +2 s.d.

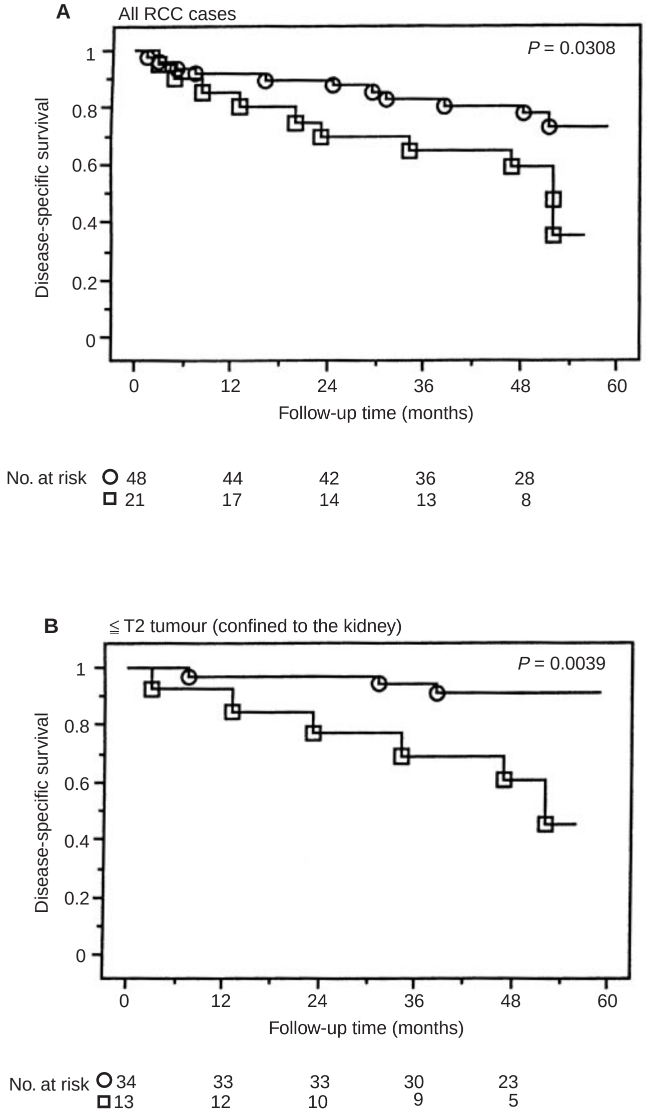

**Figure 2** (**A**) Survival of all RCC patients categorized according to the sCD95 level. (**B**) Survival of RCC patients whose tumours were confined to the kidney (⩽T2 tumour) categorized according to the sCD95 level. ○: normal sCD95 group (⩽ 2.93 ng ml$^{-1}$); □: elevated sCD95 group. The cut off level was described in Figure 1. The significance of the difference between the curves was calculated by the log-rank test

### RESULTS

Serum sCD95 levels for RCC patients (mean = 2.95 ng ml$^{-1}$) were significantly higher than those normal controls (mean = 2.14 ng ml$^{-1}$; $P < 0.001$). Twenty-one (29%) of 72 patients had elevated sCD95 (elevated sCD95 group: which was defined as more than the cut-off level, i.e. mean of healthy donors + 2 standard deviations (s.d.): 2.93 ng ml$^{-1}$; Figure 1). The correlation between pathological factors (TNM classification, histological grade or cell type) and sCD95 level was not statistically significant (Table 1).

The disease-specific survival rate was significantly lower in the elevated sCD95 group than in the normal sCD95 group by the Log rank test (Figure 2A). In each subgroup which generally have better prognosis (Lanigan, 1995), i.e. low stage (lower than T2: tumour confined to the kidney; Figure 2B), low grade (G1; data not shown), clear cell subtype (not shown) or no distant metastasis

**Table 2** Univariate and multivariate analysis of survival

| | | Univariate | | Multivariate | |
|---|---|---|---|---|---|
| Variable | Categories compared[a] | Hazard ratio (95% CI) | *P*-value | Hazard ratio (95% CI) | *P*-value |
| T category | ≤T2 vs ≥T3 | 0.255 (0.102–0.635) | 0.0034 | 0.073 (0.015–0.345) | 0.0010 |
| M category | M0 vs ≥M1 | 0.060 (0.023–0.154) | < 0.0001 | 0.013 (0.002–0.109) | < 0.0001 |
| V category | V0 vs ≥V1 | 0.460 (0.161–0.154) | 0.1469 | 1.950 (0.392–9.701) | 0.4145 |
| Histological cell type | Clear cell subtype vs other subtypes | 1.908 (0.541–6.732) | 0.3154 | 4.125 (0.799–21.288) | 0.0905 |
| Histological grade | G1 vs ≥G2 | 0.449 (0.161–1.246) | 0.1241 | 0.133 (0.021–0.820) | 0.0298 |
| sCD95 level | Normal (≤ 2.93 ng $ml^{-1}$) vs elevated | 0.401 (0.170–0.946) | 0.0368 | 0.211 (0.049–0.900) | 0.0355 |

Hazard ratios associated with pathological factors and serum sCD95 levels in 72 consecutive patients with RCC. [a]For each variable, the prognostic significance of the first category with the reference category (the second category listed). CI, confidence interval.

(not shown), the survival rate was also significantly lower in the elevated sCD95 group than in the normal sCD95 group.

Furthermore, T category, M category, grade and serum sCD95 level were shown to be significant and independent prognostic factors by univariate and multivariate analysis (Table 2).

### DISCUSSION

We previously reported that RCC cell lines (ACHN, Caki-1, Caki-2, A498, KH39 and KRC/Y) expressed CD95 antigen, and that the apoptosis-inducing anti-CD95 monoclonal antibody CH-11 induced apoptosis in ACHN cells in a dose-dependent manner (Tomita et al, 1996*a*). The cytotoxic effect of anti-CD95 antibody on certain renal cell carcinoma cell lines was augmented by pretreatment of these cells with interferon-γ (Tomita et al, 1996*a*). On the other hand, immunohistochemical analysis of resected tissues from the patients with RCC revealed stronger expression of CD95 antigen on the surface of cancer cells than that of normal cells (Nonomura et al, 1996). These results suggested that CD95 may be a possible target for novel approaches to treatment of human RCC.

The molecular mechanisms by which tumour cells evade apoptotic stimuli is thought to include intracellular mechanism and inactivation of death receptors. One of the major intracellular mechanisms to induce apoptosis is known to include expression of the anti-apoptotic molecules bcl-2 and bcl-xL (Boise et al, 1995; Tomita et al, 1996*a*, 1996*b*). Other molecules including I-FLICE/casper may inhibit apoptotic signals (Hu et al, 1997). sCD95 were shown to antagonize both anti-CD95 and CD95 ligand-mediated cell lysis (Cheng et al, 1994; Owen-Schaub et al, 1995). sCD95 has been identified in the supernatants of activated human lymphocytes and several tumour cell lines (Cheng et al, 1994; Owen-Schaub et al, 1995). For example, osteosarcoma cell line LM2-p53 expresses CD95 and is resistant to the cell killing effects of anti-CD95 (Owen-Schaub et al, 1995). The most straightforward explanation for the antagonist activity of sCD95, which is secreted from the cell line itself, is competition with cell-surface CD95 for anti-CD95 or CD95 ligand binding. Since apoptosis is known to be a critical parameter in tumorigenesis, regulation of CD95 signalling may prove to be a primary pathogenetic event in the evolution of some malignancies.

Elevated levels of sCD95 have been observed in the serum of patients with autoimmune disease (Jodo et al, 1997), haematopoietic (Knipping et al, 1995) and non-haematopoietic malignancies (Midis et al, 1996). Midis et al (1996) reported that the majority of cancer patients (including ovarian, gall bladder, lung, melanoma, head and neck etc.) examined (58 of 67) demonstrated elevated in sCD95 levels relative to normal controls. Among breast carcinoma or colon carcinoma patients, those with metastatic disease demonstrated significantly higher serum sCD95 levels than those with primary disease (Midis et al, 1996). Presence of sCD95 detected by ELISA in the present study may be derived from truncated CD95 molecules (Nagata, 1994; Tomokuni et al, 1997). Alternatively, and not mutually exclusively, CD95 was excreted by plasma membrane-derived extracellular vesicles as reported by Albanese et al (1998).

The mean sCD95 level of healthy donors (2.14 ng $ml^{-1}$) in this study was very close to 1.97 ng $ml^{-1}$, mean sCD95 level which was reported by Tomokuni et al (1997). Therefore, we consider that normal controls in the present study were reliable for comparison with RCC patients.

In the present study, serum sCD95 levels of RCC patients were shown to be significantly higher than those of healthy donors. The disease-specific survival rate was significantly lower in the elevated sCD95 group than in the normal sCD95 group. Moreover, serum sCD95 level was independent prognostic factor by multivariate analysis. Although the biological function of sCD95 in patient's serum remains to be investigated, these results suggested that evaluation of serum sCD95 level in RCC patients may be a useful indicator for prognosis.

### REFERENCES

Albanese J, Meterissian S, Kontogiannea M, Dubreuil C, Hand A, Sorba S and Dainiak N (1998) Biologically active Fas antigen and its cognate are expressed in plasma membrane-derived extracellular vesicles. *Blood* **91**: 3862–3874

Boise LH, Minn AJ, Noel PJ, June CH, Accavitti MA, Lindsten T and Thompson CB (1995) CD28 costimulation can promote T cell survival by enhancing the expression and Bcl-xL. *Immunity* **3**: 87–98

Cheng J, Zhou T, Liu C, Shapiro JP, Bruer MJ, Kiefer MC, Barr PJ and Mountz JD (1994) Protection from Fas-mediated apoptosis by a soluble form of the Fas molecule. *Science* **263**: 1759–1762

Fellenberg J, Mau H, Schhuerpflung C, Ewerbeck and Debatin KM (1997) Modulation of resistance to anti-APO-1-induced apopotosis in osteosarcoma cells by cytokines. *Int J Cancer* **72**: 536–542

Hu S, Vincenz C, Ni J, Gentz R and Dixit VM (1997) I-FLICE, a novel inhibitor of tumor necrosis factor receptor-1- and CD-95-induced apoptosis. *J Biol Chem* **272**: 17255–17257

Japanese Urological Association, Japanese Pathological Society and Japanese Radiological Society (1992) *General Rule for Clinical and Pathological Studies on Renal Cell Carcinoma*. 2nd Edition, Aso Y. (ed) Kanehara-Shuppan: Tokyo

Jodo S, Kobayashi S, Kayagaki N, Ogura Y, Feng Y, Amasaki Y, Fujisaku A, Azuma M, Yagita H, Okumura K and Koike T (1997) Serum levels of soluble Fas/APO-1 (CD95) and its molecular structure in patients with systemic lupus erythematosus (SLE) and other autoimmune diseases. *Clin Exp Immunol* **107**: 89–95

 © 1999 Cancer Research Campaign

Knipping E, Debatin K-M, Stricker K, Heilig B, Eder A and Krammer PH (1995) Identification of soluble APO-1 in supernatants of human B- and T-cell lines and increased serum levels in B- and T- cell leukemias. *Blood* **85**: 1562–1569

Lanigan D (1995) Prognostic factors in renal cell carcinoma. *Br J Urol* **75**: 565–571

Midis GP, Shen Y and Owen-Schaub LB (1996) Elevated soluble Fas (sFas) levels in nonhematopoietic human malignancy. *Cancer Res* **56**: 3870–3874

Nagata S (1994) Fas and Fas ligand: a death factor and its receptor. *Adv Immunol* **57**: 129–144

Nonomura N, Miki T, Yokoyama M, Imazuy T, Takeda T, Takeuchi S, Kanno N, Nishimura K, Kojima Y and Okuyama A (1996) Fas/APO-1-mediated apoptosis of human renal cell cancer. *Biochem Biophys Res Com* **229**: 945–951

Owen-Schaub LB, Angelo LS, Radinsky R, Warew CF, Gesner TG, Bartos DP (1995) Soluble Fas/Apo-1 in tumor cells: a potential regulator of apoptosis? *Cancer Lett* **94**: 1–8

Tomita Y, Kawasaki T, Bilim V, Takeda M and Takahashi K (1996*a*) Tetrapeptide DEVD-aldehyde or YVAD-chloromethylketone inhibits Fas/Apo-1 (CD95)-mediated apoptosis in renal-cell-cancer cells. *Int J Cancer* **68**: 132–135

Tomita Y, Bilim V, Kawasaki T, Takeda M, Takahashi K, Ismail O, Magnusson K and Wiman K (1996*b*) Frequent expression of bcl-2 in renal-cell carcinomas carring wild-type p53. *Int J Cancer* **66**: 322–325

© 1999 Cancer Research Campaign
